# Analysing the gene expression profiles of the orphan nuclear receptors NR4A1, NR4A2 and NR4A3 in premalignant lesions of the cervix and cervicitis

**DOI:** 10.1016/j.eurox.2024.100355

**Published:** 2024-11-13

**Authors:** Rosa P. Cruz-Nieves, Gladys E. Ramírez-Rosales, Javier González-Ramírez, Fausto Sánchez-Muñoz, Armando Ruiz-Hernández

**Affiliations:** a Department of Pharmacology, School of Medicine, Autonomous University of Baja California, Mexicali, Baja California, Mexico; b Department of Pathophysiology, School of Medicine, Autonomous University of Baja California, Mexicali, Baja California, Mexico; c Gynecologic Dysplasia Clinic, Medical Oncology Specialties Unit, Mexicali, Baja California, Mexico; d Molecular Biology Laboratory, Health Sciences Unit, Mexicali Campus, Autonomous University of Baja California, Mexicali, Baja California, Mexico; e Immunology Department, Ignacio Chávez National Institute of Cardiology, Mexico City 14080, México

**Keywords:** Premalignant lesions, Uterine, Cancer, Receptors, Cervicitis

## Abstract

**Objective:**

The main objective of the present study was to evaluate the expression of nuclear orphan receptors in the development of Uterine Cervical Cancer (UCC). The principal cause of dysplastic changes in cervical epithelium is the presence of the human papilloma virus leading to the development of cervical intraepithelial neoplasia I (CIN I), high-grade lesions (CIN II and CIN III) and, finally, invasive cancer. Despite the existence of various treatments and vaccines, there is still a high mortality rate. There is evidence of the participation of a group of nuclear receptors called orphans in the development of various diseases, including cancer.

**Study design:**

The expression levels of the orphan receptors NR4A1, NR4A2, and NR4A3 were measured using real-time polymerase chain reaction (RT-PCR) in samples obtained through colposcopy from forty-five patients who attended the Medical Oncology Specialties Unit (UNEME) in Mexicali, B.C.

**Results:**

Forty-five cervical biopsy results were obtained, indicating cervicitis, CIN I, or CIN III, none of them CIN II. Our results showed that orphan receptors expressed in a specific manner depending on the degree of premalignant lesions. NR4A1 overexpressed in cervicitis (p < 0.05). NR4A3 was significantly expressed in CIN I (p < 0.05) and NR4A2 was expressed in both cervicitis and CIN III (p > 0.05).

**Conclusion:**

Our data suggest, for the first time, that nuclear receptors might be involved in the various stages that precede the development of invasive UCC.

## Highlights

• First evidence suggesting nuclear receptors may contribute to the progression from cervical dysplasia to invasive cancer.
• Orphan receptors NR4A are expressed in human cervix.
• New biomarkers of premalignant cervix lesions.
• NR4A receptors participate in inflammatory processes of the cervix.

## Introduction

Uterine cervical cancer (UCC) is the fourth leading cause of cancer mortality among women of reproductive age, accounting for approximately 4.4 million deaths [1], [2]. The vast majority of deaths occur in developing countries, despite the availability of current screening methods such as cervical cytology. This fact is partly due to the high prevalence of human papilloma virus (HPV) infection, which is a necessary factor for the development of this neoplasm, though not enough, i.e., not all individuals with HPV infection will develop carcinomas [3], [4].

This pathology involves the infection of the basal cells of the cervical epithelium by HPV, particularly in the transformation zone. Viral replication in these cells causes changes that begin in the lower third of the epithelium, characterized by atypia, enlarged and hyperchromatic nuclei, and hypochromic perinuclear halo, constituting CIN I. HPV persistence can lead to progression or transformation from CIN I to CIN II and III affecting the upper third of the cervical epithelium, and subsequently, to invasive cancer [4].

Cervicitis is a prevalent diagnosis in cervical cytology. It's an inflammatory response located in the cervix that infects the cells of the columnar epithelium of the endocervical glands and the squamous epithelium of the ectocervix. This response is typically in reaction to an infectious agent, although the inflammation of the cervix is not widely understood [5], [6]. It has been recognized that inflammation influences the growth of normal cells, tumor cells, and stromal cells. Therefore, it is possible for the tumor initiation process to be influenced by the microenvironment [5]. In the case of the cervix, this relationship between inflammation and cancer has not been studied in depth. The adaptive immune system plays a crucial role in the response against the pathogens that cause cervicitis. The immune response in the cervical uterine tissue begins with antigen presentation mediated by antigen-presenting cells (APCs), which in turn activate CD4 + and CD8 + leukocytes. In CD4 + lymphocytes, it induces a Th1 profile with the subsequent release of cytokines: IL-1. IL-6, TNF alpha, and Interferon. These cytokines promote the differentiation of macrophages towards an M1 profile. On the other hand, the activation of CD8 + lymphocytes activate death mechanisms through the release of granzymes and perforins. Both lymphocytes immunological responses determine the elimination of the pathogenic agent [6].

Furthermore, several types of cancer and inflammatory diseases have been linked to changes in the expression of orphan nuclear receptors NR4A [7]. This subfamily of receptors is composed of three subtypes, namely: NR4A1 (Nurr77, TR3, NGFI-B); NR4A2 (Nurr1, NOT); and NR4A3 (NOR1, MINOR). These receptors can either exhibit tumor suppressive activity or play a prooncogenic role based on the microenvironment and tissue [8].

Moreover, NR4A receptors are present in cervical cancer cells and are viewed as prooncogenic and their expression can be regulated in HeLa cell variants. It is our understanding that NR4A receptors can participate in the development of UCC, therefore, we found that nuclear receptors might be involved in the different stages that precede the development of invasive cancer.

## Material and methods

### Recruitment of patients and sample collection

This study was approved by the Ethics and Research Committee of the Maternity Hospital of Mexicali (register number CDEI-0008–21). All participants provided informed consent before their enrollment in the study, ensuring their respect, safety and confidentiality. A total of forty-five patients from January 2022 to January 2023 at the Medical Oncology Specialties Unit (UNEME) in Mexicali, Baja California, Mexico. Fresh cervical tissue samples were collected from patients who underwent colposcopy and cervical biopsy. The cervix samples were divided into two parts, one of which was used for histopathological analysis and the other was stored in −80 °C refrigerator for gene expression analysis.

### Total RNA extraction and cDNA synthesis

Total RNA was isolated from the cervical tissues using TRIzol® Reagent (Thermo Fisher Scientific, USA), a commercially available mixture of phenol and guanidine isothiocyanate, according to the protocol described by the manufacturer. The concentration and purity of the RNA were determined by measurement of the optical densities at 260 and 280 nM using a NanoDrop 1000 spectrophotometer (Thermo Fisher Scientific, USA). An A260/A280 ratio of 1.8 was required for these studies. The RNA solutions were diluted to a working concentration of 1 µg/mL in nuclease-free water with the addition of RNase inhibitor. cDNA was prepared using the iScript cDNA synthesis kit (Bio-Rad, USA), according to the instructions of the manufacturer.

### Determination of the expression of orphan receptors

Approximately 1 µg RNA was reverse transcribed and used for real-time PCR analysis, by means of the QuantStudio™ 1 system (Thermo Fisher Scientific, USA). The final volume of the reaction mixture was 10 µL: it contained 5 µL of PanGreen Universal Master Mix (Bio-Helix Ltd, Taiwan), 0.3 µL forward primer (Integrated DNA Technologies, USA), 0.3 µL reverse primer (Integrated DNA Technologies, USA), 3.4 µL of H2O PCR grade, and 500 ng of sample. The primers for PCR were NR4A1, NR4A2, NR43 and GADPH (Table 1). The mRNA expression levels of receptors NR4A1, NR4A2 and NR4A3 were determined by means of the ΔΔCt method, with glyceraldehyde-3-phosphate dehydrogenase (GAPDH) as the normalization standard.

**Table 1:** Primer sequences used in this study.

Receptor	Primers	Sequences	(°C)
NR4A1	Fw	TTCAAAACCCAAGCAGC	50.4
	Rs	GGAAGATGCTGGGGATGTA	50.3
NR4A2	Fw	ACACACACACACACACAC	50.1
	Rs	GTTGAAGAAGGCAAAGGCT	50.3
NR4A3	Fw	ACCTATCATTTCCTGTCCTTCC	51.3
	Rs	GAGTATTGTGTTGGGGTTGTG	51.3
GADPH	Fw	AAGCCTGCCGGTGACTAAC	60
	Rs	TCGCTCCACCTGACTTCC	60

### Statistical analysis

Statistical analysis was performed using GraphPad Prism (GraphPad Software 8.0.1). All results were expressed as mean ± standard error. The mRNA relative expressions of NR4A1, NR4A2 and NR4A3 receptors were obtained by the ΔΔCt method. The data obtained from the ΔΔCt method was used to make the statistical comparisons among multiple groups with abnormal distribution and were carried out using Kruskal Wallis test. P < 0.05 were considered as statistically significant.

## Results

### Patients

In the present study, tissue samples of the cervix uteri were collected from women who attended UNEME Oncology *(n* = 45) to undergo cervical biopsy directed by colposcopy. The samples contained the cervical epithelium and its underlying stroma. The tissue samples were collected from patients aged between 16 and 55 years, with an average age of 32.3 years. Three of these patients were in the postmenopausal stage. The average age of menarche onset had been 12 years. Regarding the beginning of sexual life, it had occurred at 16 years of age, and 26 % of the women had reported more than three sexual partners. The use of contraceptive methods had been as follows: injectable hormonal (n = 1; 2.2 %); oral hormonal (n = 3; 6.7 %); intrauterine devices (n = 6; 13.3 %); condoms (n = 6; (13.3 %); and salpingoplasty (n = 15; 33.3 %). Seven (15.6 %) women had not used any method. The parity in the patients corresponded to four nulliparous (8.9 %), 12 primiparous (26.7 %), 19 multiparous (42.2 %), and 10 grand multiparous (22.2 %). Regarding other factors, five patients were smokers, 14 were alcoholics, and one patient had reported cocaine use. Regarding manifestations, 12 patients stated that they had exhibited intermittent bleeding, and six had suffered from postcoital bleeding. The categorization of the patients by body mass index indicated that 75.5 % of them had body mass indexes > 30, which was compatible with overweight and obesity (Table 2).

**Table 2:** Demographic and clinical characteristics.

Variable	n = 45	
Age (years)		
Mean	32.3	
Minimum	16	
Maximum	55	
Body mass index	No.	%
Low weight	1	2.2
Normal	10	22.2
Overweight	14	31.1
Obesity	20	44.4
Menarche (years)		
Mean	12	
Minimum	9	
Maximum	17	
Onset of active sexual life (years)		
Mean	16	
Minimum	13	
Maximum	22	
Sexual partners		
1 to 3	33	
> 3	12	
Contraceptive method		
Hormonal	9	20
Condom	6	13.3
Intrauterine device	6	13.3
Salpingoplasty	17	37.8
None	7	15.6
Pap test		
ASCUS	12	26.7
CIN I	25	55.6
CIN II	1	2.2
CIN III	3	6.7
Other	4	8.9
HPR of cervical biopsy		
Cervicitis	16	35.6
CIN I CIN II	19 0	42.2 0
CIN III	10	22.2

HPR = histopathological result; ASCUS=atypical squamous cells of undetermined significance; CIN = cervical intraepithelial neoplasia.

### Tissue samples

Among the forty-five cervical samples collected for one year, 35.6 % exhibited acute and chronic non-specific endocervicitis and exocervicitis, 42.2 % exhibited CIN I, and 22.2 % CIN III. The histopathology study did not report any result as CIN II. The patients had cervical cytology results prior to the colposcopies with biopsies. Benign findings (atrophy or inflammation) were found in 8.89 % of cases, ASCUS (atypical cells of uncertain significance) in 26.7 %, CIN I in 55.6 %, CIN II in 2.2 % and CIN III in 6.7 % of the patients. The histopathological study of high-grade CIN confirmed high-grade lesions indicated by the cytological finding in only two cases (CIN II or CIN III) (Table 3). The comparison of these data indicated that they were not statistically significant.

**Table 3:** Cervical biopsy results related to cytology results.

HPR	Results of cervical cytology				
	Benign results	ASCUS	CIN I	CIN II	CIN III
**Cervicitis**	12.5 (n = 2)	37.5 (n = 6)	50 % (n = 8)	0	0
**CIN I**	10.5 % (n = 2)	26.3 % (n = 5)	52.6 % (n = 10)	0	10.5 % (n = 2)
**CIN II**	0	0	0	0	0
**CIN III**	0	10 % (n = 1)	70 % (n = 7)	10 % (n = 1)	10 % (n = 1)

HPR = histopathological result; ASCUS = atypical squamous cells of undetermined significance; CIN = cervical intraepithelial neoplasia.

### Expression of receptors NR4A1, NR4A2 and NR4A3

**Fig. 1:**
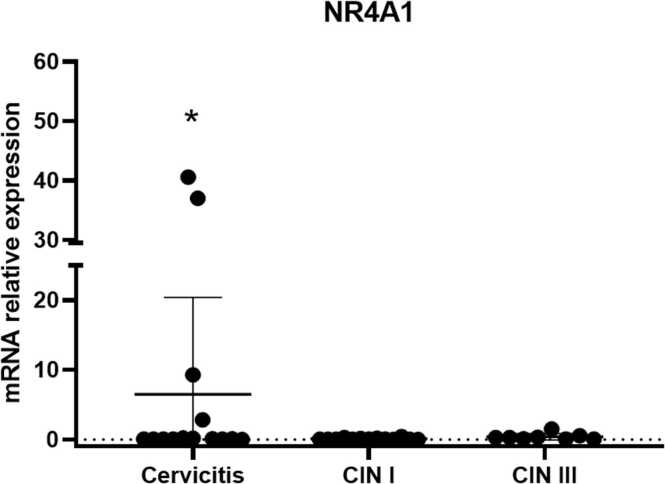
Expression of the NR4A1 receptors in premalignant lesions: Cervicitis, CIN I and CIN III. Values are expressed as mean ± standard error normalized to GAPDH. Values of *p* < 0.05 were accepted as statistically significant using the Kruskal-Wallis test.

**Fig. 2:**
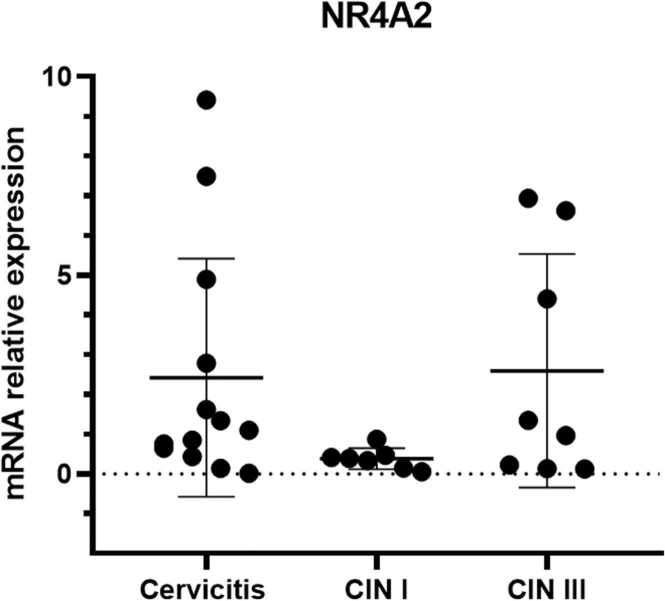
Expression of the NR4A2 receptors in premalignant lesions: Cervicitis, CIN I and CIN III. Values are expressed as mean ± standard error normalized to GAPDH. Values of *p* < 0.05 were accepted as statistically significant using the Kruskal-Wallis test.

**Fig. 3:**
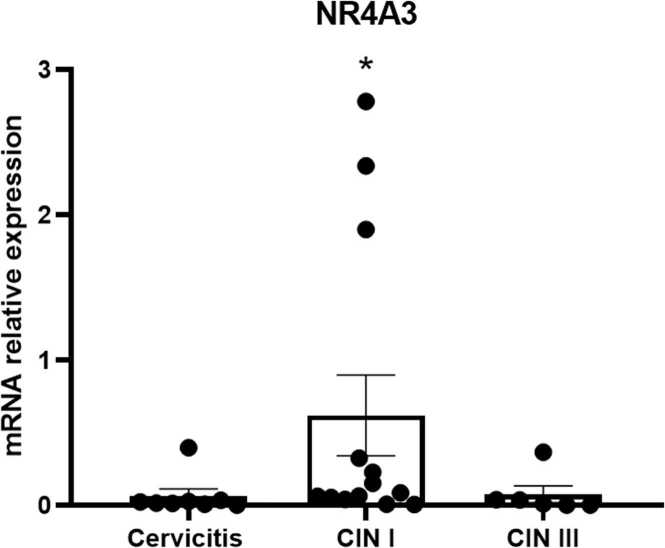
Expression of the NR4A3 receptors in premalignant lesions: Cervicitis, CIN I and CIN III. Values are expressed as mean ± standard error normalized to GAPDH. Values of *p* < 0.05 were accepted as statistically significant using the Kruskal-Wallis test.

Orphan receptors NR4A1, NR4A2, and NR4A3 were expressed in cervical tissues. It was observed that the receptor NR4A1 was significantly overexpressed in cases of cervicitis when compared to CIN 1 and CIN 3 (*p* = 0.0378) (Fig. 1). On the other hand, the receptor NR4A2 was expressed in cervicitis and CIN III, with a tendency to underexpression in CIN I, a result that was not statistically significant (Fig. 2). Regarding the orphan receptor NR4A3, a significant increase in its expression was found in patients with CIN I when compared with cervicitis and CIN III (*p* = 0.0366) (Fig. 3).

## Discussion

The present study describes the analysis of NR4A receptor gene expression in cervical samples with premalignant lesions or cervicitis for the first time. These samples were obtained from patients who underwent cervical biopsies guided by colposcopy due to previous alterations in cervical cytology screening results. The NR4A1, NR4A2, and NR4A3 receptors constitute a subfamily of nuclear receptors that can be activated by peptide hormones, growth factors, cytokines, physiological stimuli, cellular stress, and inflammation [11]. Once activated, these receptors, modulate gene expression resulting in physiological and pathological phenomena such as inflammation and, later, cancer [8]. The mechanism by which these receptors regulate gene expression involves forming monomers or heterodimers. They bind directly to the promoter regions of genes where a response element is found. This results in an increase or decrease in the expression of genes that control signaling pathways related to chronic inflammation and cancer [9], [12]. Therefore, the levels of expression, cellular location, and cellular environment influence the tissue-specific expression and function of the receptors of this family [9].

The vast majority of the patients exhibited cervicitis, others CIN I, and, to a lesser extent, CIN III. Unfortunately, no samples were obtained from patients with CIN II. The mean age of the patients with cervicitis was 32.3 years, whereas that of the patients with CIN I was 34.9 and with CIN III 29.8. Such result indicates a population affected at younger ages compared to those populations assessed in other studies in the field, in which CIN I had been found at 41 years and CIN III at 45.8 years. This fact was probably due to living in a country with high rates of HPV infection, to which women are exposed earlier [9]. On the other hand, in the present study, it was also observed that these patients shared various risk factors that could lead to the onset of UCC, such as the early start of active sexual life, with a mean of 16 years, and the lack of use of a barrier contraceptive method, since only 13.3 % reported using them. Based on the anthropometry of the patients, a large part of the population of the present study had increased BMI, with 31.1 % classified as overweight and 44.4 % as obese. These data may explain the variation that existed between the results of cervical cytology and the biopsy results, since there was a negative effect of obesity on screening tests due to several factors, one of which was the technical difficulty of sample collection [10].

Regarding gene expression, there was an increase in the expression of NR4A1 in women with cervicitis compared to women who exhibited CIN I and CIN III. NR4A1 mRNA is usually rapidly, but transiently induced by multiple stimuli, while protein expression is delayed and more persistent, one of the pathways that has been identified is through NFkB signaling to mediate the inflammatory response of the immune system [11], so we propose that the increase in the expression of NR4A1 could be stimulating the inflammatory response in immune cells such as lymphocytes and macrophages of the cervical tissue, since its overexpression has been correlated to an acute inflammatory response in various tissues, it has been described that NR4A1 has an ambivalent role in inflammatory processes, promoting the response in acute processes but regulating the long-term response [12].

Various studies have reported that the disordered function of stromal cells, the alteration in cell signaling, and the secretion of paracrine factors have been related to inflammation and, consequently, to the onset of various types of cancers, including UCC. Moreover, the infiltration of immune cells into the cervical microenvironment in non-tumoral cervical cells, CIN I, II, or III may have fundamental differences that can lead to regression or progression to cancer [12], [13].

There were no significant changes with respect to the receptor NR4A2, although a tendency to overexpression was observed in cervicitis and CIN III in contrast to NR4A3, which was significantly expressed in CIN I but not in cervicitis or CIN III, a fact that could be explained by functional redundancy exhibited by these receptors [14]. This functional homology has been proposed because, in the case of NR4A1 dominant-negative transgenic mice, it lacks a phenotype, proposing that the function of this lacking receptor is being replaced by NR4A3, however, the degree of compensation varies according to the context [15].

Additionally, there is evidence that NR4A nuclear receptors are capable of inducing cellular changes and modify the cellular microenvironment, generating inflammatory responses, angiogenesis, and altering the immunity of cancer cells, which probably explains the increase in gene expression observed in the tissues analysed in the present study. This way, it should be considered that these receptors participate not only in the development of cancer but also in the development of premalignant lesions [15], [16].

## Conclusion

In summary, we concluded that the orphan receptors NR4A1, NR4A2 and NR4A3 were expressed in the cervix and that their expression could be modified in contexts of inflammation or in premalignant lesions of the cervix. It should be considered that they participate in the mechanisms involved in inflammation and the development of cervical cancer.

## CRediT authorship contribution statement

**Javier González-Ramírez:** Project administration. **Gladys E. Ramírez-Rosales:** Formal analysis, Data curation. **Rosa P. Cruz Nieves:** Writing – original draft, Investigation. **Armando Ruiz-Hernández:** Writing – review & editing, Methodology. **Fausto Sánchez-Muñoz:** Supervision.

## Declaration of Competing Interest

The authors declare that they have no known competing financial interests or personal relationships that could have appeared to influence the work reported in this paper.
